# Cognitive Training for Mild Traumatic Brain Injury and Posttraumatic Stress Disorder

**DOI:** 10.3389/fneur.2020.569005

**Published:** 2020-11-26

**Authors:** Kristin W. Samuelson, Krista Engle, Linda Abadjian, Joshua Jordan, Alisa Bartel, Margaret Talbot, Tyler Powers, Lori Bryan, Charles Benight

**Affiliations:** ^1^Department of Psychology, National Institute for Human Resilience, University of Colorado Colorado Springs, Colorado Springs, CO, United States; ^2^Department of Psychiatry, University of California San Francisco, San Francisco, San Francisco, CA, United States

**Keywords:** cognitive training, mild TBI, PTSD, memory, cognitive rehabilitation

## Abstract

Although there is evidence of mild cognitive impairments for many individuals with mild traumatic brain injury (mTBI) and posttraumatic stress disorder (PTSD), little research evaluating the effectiveness of cognitive training interventions has been conducted. This randomized controlled trial examined the effectiveness of a 9-h group cognitive training targeting higher-order functions, Strategic Memory Advanced Reasoning Training (SMART), compared to a 9-h psychoeducational control group in improving neurocognitive functioning in adults with mTBI and PTSD. A sample of 124 adults with histories of mild TBI (*n* = 117) and/or current diagnoses of PTSD (*n* = 84) were randomized into SMART (*n* = 66) or Brain Health Workshop (BHW; *n* = 58) and assessed at three time points: baseline, following training, and 6 months later. Participants completed a battery of neurocognitive tests, including a test of gist reasoning (a function directly targeted by SMART) as well as tests of verbal, visual, and working memory and executive functioning, functions commonly found to be mildly impaired in mTBI and PTSD. The two groups were compared on trajectories of change over time using linear mixed-effects models with restricted maximum likelihood (LMM). Contrary to our hypothesis that SMART would result in superior improvements compared to BHW, both groups displayed statistically and clinically significant improvements on measures of memory, executive functioning, and gist reasoning. Over 60% of the sample showed clinically significant improvements, indicating that gains can be found through psychoeducation alone. A longer SMART protocol may be warranted for clinical samples in order to observe gains over the comparison group.

## Introduction

Approximately 1.7 million traumatic brain injuries (TBI) occur in the United States each year (1, 2). The majority of those (75%) are mild traumatic brain injuries (mTBI), which often involve physical, cognitive, and affective symptoms in the acute phase followed by resolution of symptoms after ~1 month (3). However, an estimated 10–20% of patients continue to report symptoms that persist months to years after the injury (4, 5) that have been associated with social and occupational dysfunction, including under-employment, low income, and marital problems (6–9). As such, identifying efficacious interventions for cognitive deficits related to mTBI is a priority.

In addition, mTBI is highly comorbid with posttraumatic stress disorder (PTSD), which represents a potential complicating factor in recovery. Among veterans with histories of TBI, rates of PTSD range from 33 to 65% (10, 11). PTSD has been associated consistently with mild neurocognitive deficits in a number of domains. Meta-analyses reveal significant differences between individuals with PTSD compared to healthy and trauma-exposed controls, representing medium to large effect sizes, in the domains of verbal learning and memory, processing speed, attention/working memory, and executive functions (12, 13). Moreover, patients with PTSD self-report cognitive problems with detrimental impacts on social and occupational functioning (14–16).

Research on neuropsychological functioning in mTBI is less consistent, in part due to the heterogeneity in the criteria used to define mTBI, populations sampled, time since injury, and mechanisms of injury. Individuals with persisting post-concussive cognitive complaints have shown impairments in sustained attention (17–19), divided attention (20), selective attention and inhibitory control (17, 21), cognitive flexibility and planning (8, 22, 23), processing speed (24), verbal memory (25–28), and visual memory (18). In addition, even patients who report full recovery may continue to experience cognitive problems under conditions of physical or psychological stress (29). The high comorbidity of mTBI and PTSD presents the potential for greater impaired functioning. In studies examining mTBI and PTSD concurrently, the majority found that while PTSD was related to neuropsychological impairments, mTBI was not (30–32). However, some studies have found a poorer performance profile in individuals with both mTBI and PTSD, as compared to those with mTBI or PTSD alone (21, 31). Given the overlap of structural and functional changes and neurocognitive deficits seen in both PTSD and mTBI [e.g., (33, 34)] there is a critical lack of investigations that evaluate cognitive rehabilitation approaches for these individuals. This paper attempts to fill this void.

Brain regions particularly vulnerable to both mTBI and PTSD are the frontal lobes, which are involved in learning and memory operations, executive functioning, attention and working memory, and reasoning abilities. The importance of frontal lobe function in neurological recovery after TBI is reflected in functions such as motivation, attention, and working memory that are prerequisites for optimal rehabilitation. Difficulties in these areas are considered poor prognostic indicators for TBI rehabilitation (35). Rehabilitation of frontal lobe functions is thus a crucial goal for enhancing recovery from brain injuries.

Prior studies have demonstrated that training-based rehabilitation therapy helps patients with neurological damage (36–39). However, a major limitation of many rehabilitation studies is the lack of a theoretical foundation based on known mechanisms of brain function, which can serve to guide treatment development. The proliferation of computer-based technology over the past decade has led to the rise of the rehabilitative models that employ repeatable tasks and mass training. Despite their popularity, results on the efficacy of these restorative training programs have been mixed, and considerable debate remains regarding how to effectively restore cognitive capacities following TBI.

To date, randomized controlled trials (RCTs) aimed at improving cognitive functioning in patients with mTBI have shown limited effectiveness (40–42). The research literature examining cognitive rehabilitation (CR) for mTBI has been limited by a lack of well-designed and sufficiently powered studies that fail to include control groups and functional outcomes (41, 43). RCTs aimed at treating cognitive symptoms in the post-acute or chronic stage are particularly lacking. A recent exception is a study that compared psychoeducation, computerized brain training, therapist-led CR, and a therapist-led CR/psychotherapy hybrid (40). The four interventions were equivalent in improving cognitive functioning, with between 23 and 33% of participants showing reliable change on the primary working memory outcome. The therapist-led CR and the integrated groups showed significantly greater improvements on a self-report of functional cognitive and behavioral difficulties (23 and 19%, respectively, in the two groups, showed reliable change) compared to psychoeducation and computerized brain training. However, these interventions were resource-intensive, with treatment consisting of daily therapy for 6 weeks.

Research examining CR for PTSD-related cognitive impairments is lacking. Recently, researchers tested the effectiveness of a computerized cognitive training program, a hybrid of Lumosity and MyBrainSolutions, in improving neurocognitive functioning in a sample of primarily motor vehicle accident survivors recruited from emergency rooms (44). Compared to the control group that engaged in computer games, card games, and matching tasks, the CR group showed significant improvements (Cohen's *d* = 0.58) in cognitive flexibility after 1 month of CR, assessed 3 months following the trauma. This study lends preliminary support for the use of cognitive training for PTSD, particularly in the acute phase, although less is known about the treatment of long-term cognitive impairments related to PTSD.

Researchers have argued that for rehabilitative interventions to be successful, they must target skills that are directly applicable to daily functioning, particularly for patients with more mild impairment levels, as is the case with mTBI and PTSD (45, 46). In addition, given the importance of frontal lobe functioning in both mTBI and PTSD, cognitive training must address higher-order, frontal lobe-mediated cognitive skills.

The development of Strategic Memory and Reasoning Training [SMART; (47, 48)] addressed this need, with the goal of targeting higher-order functions found to be crucial for the recovery following brain injury (49). Prior research has shown that when these specific brain functions are targeted, such as the ability to focus on a task while ignoring irrelevant information, brain changes are more significant (49–51). SMART emphasizes top-down processing by targeting focused attention, assimilation of information, mental flexibility, and innovation, all higher-order cognitive functions driven by the frontal lobes. Other top-down cognitive training programs have demonstrated effectiveness in improving cognitive and daily functioning in individuals reporting more severe brain injuries (50–53); however, limited research has been devoted to milder brain injuries.

The goal of SMART is to teach metacognitive strategies to enhance time and cognitive resource management through goal setting and the inhibition of distracting or irrelevant stimuli. In addition, it prioritizes deeper level synthesis of information to obtain the “gist” while encouraging fluid and flexible thinking (54, 55). Training in gist reasoning, or “the ability to strategically comprehend and convey generalized, core meaning(s) from complex information,” is a primary component of the SMART protocol [54, p. 2]. Strong gist reasoning minimizes the cognitive overload of competing stimuli in the environment and focuses on constructing meaning rather than remembering details. Gist reasoning impairments have been found in adults and adolescents with mild and moderate TBI (56, 57). In addition, gist reasoning is associated with frontal lobe activation and draws upon functions of inhibitory control, working memory, cognitive flexibility, abstract reasoning, and fluency (56, 58), domains often impaired in both TBI and PTSD.

The effectiveness of SMART has been tested in a number of studies of adults and adolescents with TBI. The typical SMART training consists of 15 h of training conducted over 10 group sessions in the first 5 weeks and a final 3 h of training at spaced intervals over the next 3 weeks. Vas et al. (59) conducted an RCT comparing SMART to a psychoeducational control (Brain Health Workshop; BHW) in adults with TBI histories of >2 years and moderate functional impairment. The majority of participants' brain injuries were not specified as mild, moderate, or severe. SMART was associated with significantly greater improvements in gist reasoning compared to psychoeducation controls. Generalized improvements were also seen in working memory and participation in functional activities, domains that were not directly targeted by the SMART training. These gains were maintained 6 months post-training.

A subsequent study with children and adolescents who had received a mild, moderate, or severe closed-head TBI at least 6 months prior to study participation also demonstrated positive findings. These participants, who demonstrated below average gist reasoning skills at baseline, completed either a shorter SMART training protocol of eight 45-min sessions or a memory training (60). The SMART participants displayed significant improvements in their ability to abstract meanings (*d* = 1.41) and recall facts (*d* = 0.77) compared to the control group. The SMART participants also demonstrated significant improvements in the untrained executive functions of working memory (*d* = 0.94) and inhibition (*d* = 0.73), whereas the control group participants did not. In a larger RCT of adults with a history of unclassified TBI who were experiencing mild cognitive impairments at the time of the training, Vas et al. (57) compared receiving at least 18 h of SMART to BHW over 8 weeks. They found greater improvements for SMART participants on measures of gist reasoning, set shifting, and self-reported psychological health and daily function. These studies demonstrate the effectiveness of SMART in samples of individuals with a range of brain injury severity. One of the purposes of the present study was to assess its effectiveness in a sample of adults with milder brain injuries.

Notably, SMART is also effective in improving cognitive functioning in cognitively healthy individuals (54, 58, 61–63), which suggests that SMART may show benefits for individuals with mTBI and PTSD who have less impaired, or even average, functioning. Lack of impairment is not uncommon for many individuals with mTBI or PTSD [e.g., (25, 64–70)], yet appraisals of cognitive functioning are often negative and not aligned with objective performance (16, 71–74). As a result, targeting cognitive functions via an approach that emphasizes neuroplasticity and psychoeducation may additionally improve expectancies and appraisals.

The developers of SMART recently introduced a shortened SMART training of three, 3-h sessions that has not yet been tested with mTBI. Similarly shortened protocols have shown gains in higher-order reasoning, working memory, and immediate and delayed memory in adolescents and adults with chronic mTBI (75). To our knowledge, SMART has never been tested with patients with PTSD, a population that struggles with cognitive problems with limited existing cognitive rehabilitation research. The overlap of both structural and functional changes and neurocognitive deficits seen in both PTSD and mTBI [e.g. (33, 34)] and the high rates of comorbidity associated with poorer functional outcomes, highlights the need for cognitive rehabilitation research that addresses both conditions alone and together. The purpose of the current study was to investigate the effectiveness of a shortened SMART training program, compared to a psychoeducation control, in improving neurocognitive functioning in patients with mTBI and/or PTSD. We hypothesized that participation in SMART, compared to the control group, would result in improved gist reasoning as well as improved performance on tests of generalized cognitive functions (working memory, verbal memory, visual memory, and executive functioning).

## Methods

### Participants

In the present study, 144 adults between the ages of 18 and 65 were recruited from several sources within the community. Nineteen percent of the sample was recruited through a registry of community members interested in trauma research. Sixty-one percent of participants responded to flyers at community clinics, military installations, and on Craigslist postings and 20% from postings of flyers and in psychology classrooms at a mid-sized Western university. Recruitment ads described that the research involved a cognitive training study or adults with TBI and/or trauma histories.

Inclusion criteria were English-speaking adults between the ages of 18 to 65 years. We allowed for diagnosis of either mild or moderate TBI [as defined by Ohio State University TBI Identification Method; OSU TBI-ID; (76)] and/or diagnosis of subthreshold or full PTSD [as determined by Clinician-Administered PTSD Scale for DSM-5; (77)]. Exclusion criteria included self-report on a phone screen interview of pre-existing cerebral palsy, intellectual disability, autism, epilepsy, psychotic disorder or bipolar disorder, current alcohol, or drug dependence within the last 3 months, stroke, or pervasive developmental disorder. Participants who denied exclusion criteria and had reported a past head injury with loss of consciousness or alteration in consciousness or a psychological trauma during a phone screen were invited to participate in an in-person eligibility visit. Further exclusion criteria at this visit included poor effort on a symptom validity measure [Test of Memory Malingering; TOMM; (78)] and involvement in neuropsychological testing or cognitive training in the past 3 months as this could introduce practice effects. Participants were asked to refrain from using alcohol or non-prescription drugs, including marijuana unless medically prescribed, on days of testing. We did not require clinical impairment on neurocognitive tests (defined as 1.5 standard deviations below the mean) or self-reported cognitive problems.

**Figure 1:**
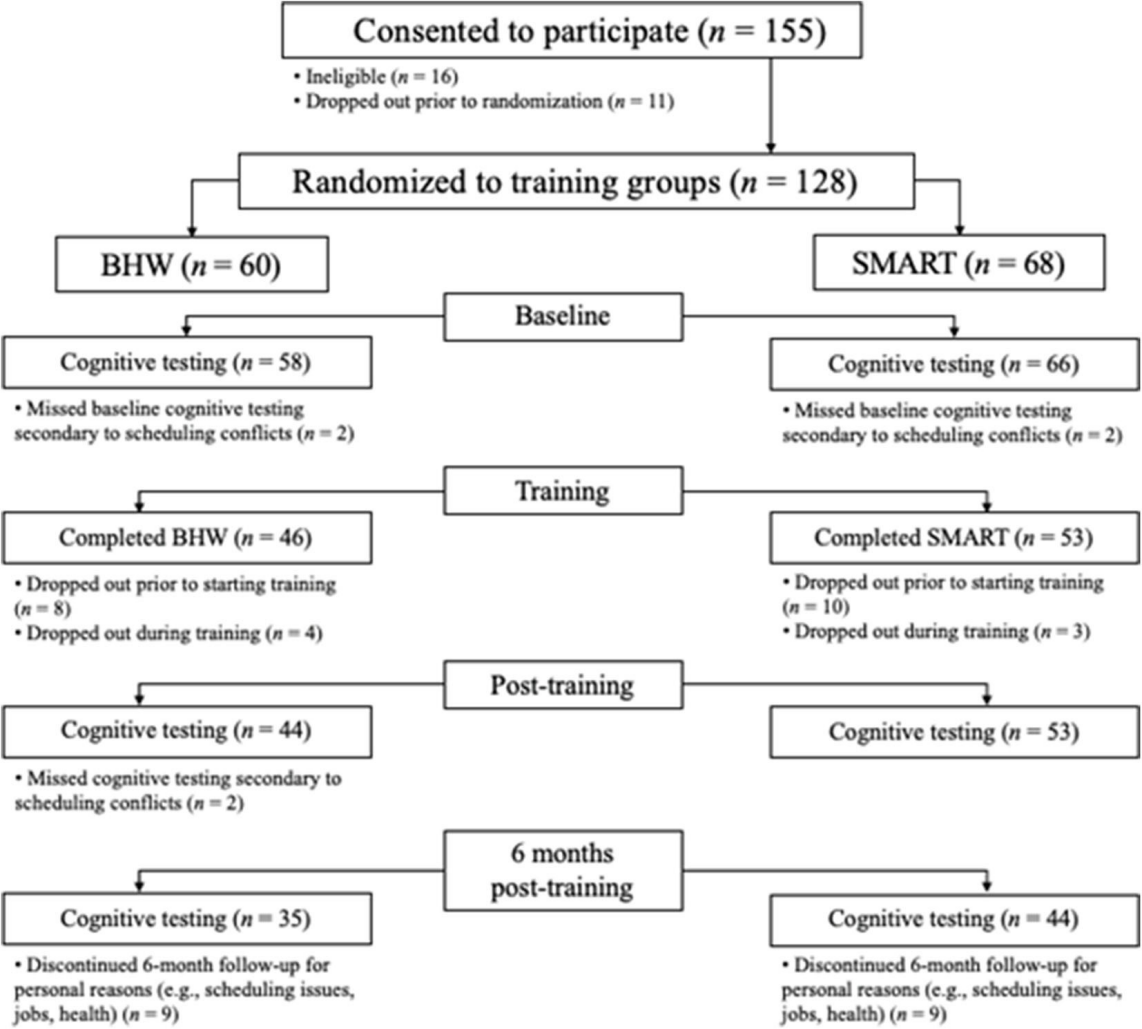
Consolidated Standards of Reporting Trials (CONSORT) flow diagram. Drop-outs in each group were due to random factors.

Figure 1 provides the Consolidated Standards of Reporting Trials (CONSORT) flow diagram of participation recruitment and completion. Of the 144 participants consented and assessed for eligibility, 128 met eligibility criteria and were randomized to training groups. Two participants from each group dropped out prior to the pre-training assessments. Eight BHW and 11 SMART participants dropped out before beginning training, and four BHW and three SMART participants dropped out during training. Forty-four BHW and 53 SMART participants completed post-training assessments and 6-month follow-up data was collected for 81% of the sample who completed post-training assessments. The following characteristics were observed in the final group of 124 participants.

#### Mild and Moderate TBI

Assessed with the Ohio TBI Identification Method [OSU TBI-ID; (76)], 95% of participants reported a history of at least one mild (*n* = 96, 82%) or moderate (*n* = 21, 18%) TBI incurred from either a single event (e.g., car accident, recreational accident; endorsed by 79%) or a repeated event (e.g., sports collisions, blast exposure, etc., endorsed by 31%). Twenty-one percent (*n* = 82) of events reported were incurred during motor vehicle accidents; 26% (*n* = 104) were sports-related accidents or injuries; 12% (*n* = 46) resulted from the person being physically assaulted; 12% (*n* = 48) of the injuries were related to military activities (e.g., blast injuries, IED explosions); and 29% (*n* = 115) involved other types of accidents (e.g., falls, hitting head on stationary objects).

At baseline, participants who reported moderate TBIs were not significantly different from the other participants on the cognitive measures of interest (*p* =.054 to.871) apart from a measure of cognitive postconcussive symptoms (Neurobehavioral Symptom Inventory). Those with moderate TBI endorsed more severe post-concussive symptoms (*M* = 8.95, *SD* = 4.02) than those with only a mild TBI (*M* = 6.67, *SD* = 3.82), *t*(113) = −2.36, *p* =.02.

#### PTSD and Subthreshold PTSD

The Clinician-Administered PTSD Scale for DSM-5 [CAPS-5; (77)], is a structured interview of the 20 diagnostic criteria for PTSD. A diagnosis of full PTSD is assigned if a participant receives a “moderate” severity and sufficient diagnostic criteria (at least one re-experiencing symptom, one avoidance symptom, two symptoms involving negative alterations in mood or cognitions, and two hyperarousal symptoms). Subthreshold PTSD is assigned if the participant meets diagnostic criteria for the re-experiencing symptom cluster and at least two other symptom clusters. Of those participants who endorsed trauma exposure (93% of the total sample), 51% (*n* = 63) met criteria for PTSD, and 17% (*n* = 21) met criteria for subthreshold PTSD. The remaining 32% of the sample who endorsed trauma exposure did not meet criteria for either subthreshold or full PTSD but were included because they met criteria for mild or moderate TBI.

### Procedure

The study protocol was approved by the Institutional Review Board at University of Colorado Colorado Springs and informed consent was obtained from all participants. Interested participants first completed a phone screen interview to determine potential eligibility. If eligible at that stage, they were invited for an initial study visit for an interview and testing. To verify eligibility, participants were administered the OSU TBI-ID (76) for TBI, the CAPS-5 for PTSD, and the TOMM (78, 79) by trained graduate students in clinical psychology. At a second visit, eligible participants were administered a battery of neuropsychological assessments that established the baseline measures for the study. Total testing took approximately 5 h. Training was initiated within 1 month of the baseline assessment and post-training assessments were conducted within a month of completion of the training. Follow-up assessments were scheduled within 6 to 7 months after training ended. Participants were compensated for each assessment visit.

The study was a double-blinded randomized control trial where both participants and examiners were blinded to treatment condition. Following the baseline eligibility interview, participants were randomly assigned to either SMART or BHW. Participants were informed that the goal of the study was to compare the effectiveness of two cognitive training programs for adults with TBI and/or PTSD symptoms.

### Training Protocols

#### SMART

SMART was delivered in small groups (*n* = 2–8) consisting of two 3-h sessions over 2 days, followed by one 3-h session a month later. Two clinical psychologists who received extensive training from SMART developers delivered the interventions. SMART strategies were introduced in a PowerPoint format and reinforced in each session. Newspaper articles, stories, pictures, and audio or video clips were used to illustrate each strategy, and the application of strategies in daily life was emphasized. Overall, sessions focused on strategic attention, integrative reasoning, and cognitive control functions (48). Homework is assigned to practice skills between sessions. Typically, the longer SMART protocols consist of initial sessions of skills training with the 1-month follow-up session being a “booster session” to review material covered. Due to the shortened protocol and perceived level of impairment of the current sample, we modified the training such that all sessions included skills training with a briefer review at each session.

#### Brain Health Workshop (BHW)

The BHW training has been used in multiple studies as a comparison training program in cognitive training trials (52, 57, 59, 80). It consists of three, 3-h sessions of fact-based information about the brain but does not train cognitive strategies. Similar to SMART, topics are introduced in a sequential manner via PowerPoint. Topics include neuroanatomy, neuroplasticity, and effects of TBI on cognitive functioning. Other sessions focus on diet, exercise, sleep, and social functioning and the relationship to brain health. Participants were encouraged to share how the topics impact their lives. Participants were given take-home reading materials on related topics that were then discussed at the last session. At home, they were instructed to watch assigned videos but had no other homework. BHW has been found to be equally engaging as the targeted treatment in prior studies [e.g., (81)].

### Measures

#### Pre-treatment Assessment Measures

At the eligibility interview, the trained clinical evaluator assessed lifetime TBI history with the OSU TBI-ID, a structured interview. The interview requires the participant to recall all injuries involving a blow to the head or neck, fall, blast exposure, or vehicular accident that may cause an injury to the brain. For each injury, the nature of altered consciousness is assessed. The OSU TBI-ID demonstrates strong interrater reliability and predictive validity (76). Participants are classified as sustaining an mTBI if loss of consciousness (LOC) or alteration in consciousness for all injuries was <30 min, and moderate TBI if any injury involved a LOC between 30 min and 24 h.

In addition to the CAPS-5 to assess PTSD symptoms, participants completed the TOMM to assess cognitive effort and were excluded from the study if they scored below 45 on Trial 2, a cutoff shown to detect insufficient effort (82). The TOMM is a widely used, psychometrically sound measure of effort (83).

#### Repeated Outcome Measures

Participants completed the following measures pre- and post- training and 6 months after the final cognitive training session. We chose neuropsychological tests based on the research literature regarding deficits seen in mTBI and PTSD. Many of the measures selected were identified by the TBI Clinical Trials Network group (84) as the recommended outcome measures for TBI treatment trials and are National Institute of Neurological Disorders and Stroke (NINDS) Common Data Elements. With the exception of the Test of Strategic Learning, the neuropsychological tests assessed functions not directly targeted by the SMART training, allowing for generalizability of cognitive improvement.

To assess working memory, sustained attention, and divided attention, we administered the Paced Auditory Serial Addition Test [PASAT; (85, 86)]. Participants were asked to listen to an audio tape presenting a series of single-digit numbers and then state aloud the sum of the number more recently presented plus the number preceding it. There were two 60-item trials with interval times between numbers decreased between the trials. Prior research has shown strong split-half reliability and test-retest reliability (87). We also administered the Digit Span test of the Wechsler Adult Intelligence Scale—Fourth Edition [WAIS-IV; (88)]. Digit Span requires participants to repeat digits forwards and backwards and to repeat sequences in ascending order; the combined score of these three tasks was used.

To assess sustained attention, vigilance, inhibitory control, and inattention, the computer-based Conners' Continuous Performance Test 3rd Edition [CPT-3; (89)] was administered. This task lasts 14 min and consists of 360 trials. Respondents are instructed to not hit a computer key each time they see the letter X, but to hit a key when they see any non-X letter. Different letters are shown throughout the task at different rates of speed. Errors of omission (missing the key hit in response to non-X letters) and commission (incorrectly hitting a key to the letter X) are tracked. The CPT-3 has strong psychometric properties (89).

To assess processing speed and visual-motor coordination, the Digit Symbol and Symbol Search tests, which comprise the Processing Speed Index of the WAIS-IV (88) were used. Both measures require the test taker to reproduce or scan and match symbols quickly, efficiently, and accurately. All of the WAIS-IV tests have strong psychometric properties, including with TBI samples (90).

The California Verbal Learning Test—Second Edition [CVLT-II; (91)] was administered to assess verbal learning and memory. Participants hear a word list over five trials and repeat back as many words as they can remember; the sum of trials 1–5 serves as a measure of immediate verbal memory, and the delayed memory for the words 20 min later serves as a measure of delayed verbal memory. In addition, the Logical Memory task of the Wechsler Memory Scale, 4th edition [WMS-IV; (92)] was used to assess verbal learning and memory in a narrative context. Participants were presented with a short story and asked to repeat back as much of the story as possible immediately after. The sum of the number of details from both stories that participants are able to recall after a 20-min delay was used as a second measure of delayed verbal memory.

The Brief Visuospatial Memory Test-Revised [BVMT-R; (93)] is a measure of immediate and delayed visuospatial memory. The participant must memorize a series of designs over three trials and recreate them from memory, both immediately following display and after a delay of 20 min.

Strategic learning was examined with the Verbal Selective Learning Task (VSLT), which evaluates the ability to inhibit less important information (i.e., filtering) while focusing on more important information (i.e., prioritizing) using word lists. It was adapted from Castel et al. (94) and advanced by Hanten et al. (95, 96). The VSLT has shown sensitivity in examining strategic learning ability in children/adolescents with TBI (95, 96). For this task, the participant is asked to view a series of words on a screen. He/she is presented with 3 consecutive lists of 16 words, printed in either all capital letters or all lowercase letters. For each list, the different variations are given point values (e.g., the words in capital letters are worth 10 points for each one recalled, while the lowercase words are worth 1 point each, or vice versa). The goal is to score as many points as possible by recalling words from each list. The purpose of the task is not solely focused on how much the individual can remember, but rather if he/she is implementing a strategy to manage the memory load as efficiently as possible (i.e., prioritizing the high-value items and filtering/blocking the low-value items). The value assigned to each word was revealed to the participant prior to the presentation of each list. The initial letter format-value pairing was counterbalanced across the subjects, and the order of presentation of the lists was randomized for each participant.

Four tests were administered to assess component of executive functioning. The Delis–Kaplan (D-KEFS) Color-Word Interference Test [CWIT; (97)] measures inhibitory control and cognitive flexibility with four conditions. The first two conditions are relatively straightforward word-reading (e.g., read the word “red”) and color-naming (e.g., name the color “red”) tasks. In Conditions 3 and 4, the task becomes more complicated as color words (e.g., “red”) are printed in non-corresponding (e.g., green) ink. In Condition 3, the respondent must verbalize the color the word is printed in. In Condition 4, respondents must switch between reading the color the word is printed in or reading the word. Condition 3 performance, assessing interference, was the variable used in analyses. The Delis-Kaplan (D-KEFS) Verbal Fluency Test [VF; (97)] assesses verbal fluency with three conditions: letter fluency, category fluency, and category switching. In each task, respondents must state as many words as in 1 min following appropriate letter, category, and category switching prompts. The D-KEFS Trail Making Test [TMT; (97)] consists of five conditions. Three conditions require respondents to connect circles in the appropriate numerical (e.g., 1, 2, 3), alphabetical (e.g., A, B, C), or switching (e.g., 1-A, 2-B, 3-C, etc.) orders as instructed. Two other conditions require participants to isolate one number from others and connect circles as quickly as possible. The variable of interest for the present study was switching condition performance. Finally, inhibitory control was assessed using the CPT-3 number of commissions, in which respondents incorrectly hit a computer key in response to the letter X.

To assess gist reasoning, the skill specifically trained via SMART and thus a proximal measure of SMART efficacy, we used the Test of Strategic Learning [TOSL; (98)]. The TOSL involves synthesizing gist meaning from complex information. Participants read a complex passage and are instructed to generate a high-level summary of what they read. The summary abstraction score, the high-level lessons score, and the detail total score were computed based on a manualized objective scoring system. The summary abstraction score reflects the total number of accurately abstracted meanings from the reading; the high-level lessons score measures the number of high-level lessons the participants gleaned from the story; and the detail total score measures participants' memory for the story's detail-based information. Three different versions of the TOSL were administered at the three assessment periods using counter-balanced ordering across participants. Two trained scorers blinded to participant group status independently scored the summaries. Prior inter-rater reliability scores for the CBH raters assessed via intraclass correlation coefficients is over 90% (57).

Finally, we assessed self-report of postconcussive symptoms at all timepoints using the Neurobehavioral Symptom Inventory [NSI; (7)], a 22-item self-report measure of postconcussive symptoms. We specifically examined the Cognitive subscale of the NSI at all timepoints. The NSI is a Department of Defense core TBI outcome measure and measure and was used as a primary functional outcome for a recent large cognitive rehabilitation trial for mTBI (40).

### Data Analysis

Groups were first compared on baseline characteristics through Mann-Whitney U tests for continuous variables and Pearson Chi-Square tests for nominal/categorical variables. In order to reduce the number of statistical comparisons on neurocognitive variables (with the exception of the TOSL), a Principal Components Analysis (PCA) was conducted. The number of components to retain was identified through Parallel Analysis, a Monte Carlo simulation procedure (99). Components were retained if they exceeded the 95th percentile Eigenvalue in order to prevent over-extraction of components. Promax (oblique) rotation was used to allow components to correlate. Components were standardized from baseline values, such that subsequent timepoints were also standardized according to means and standard deviations from the baseline assessment. NSI Cognitive subscale scores were standardized using the same method.

Groups were compared on both the PCA-derived components as well as a separate analysis with the TOSL, the measure gist of reasoning. All analyses conducted using the intent-to-treat (ITT) principle, meaning that all randomized subjects were included in the analyses. Groups were compared on trajectories of change over time via linear mixed-effects models with restricted maximum likelihood (LMM). LMM are ideal for longitudinal analyses due to their ability to tolerate missing observations, assuming that they are missing at random (MAR). Although the MAR assumption cannot be formally assessed, a pattern-mixture model, which is a sensitivity analysis that examines whether patterns of missing data affect parameter estimates, was used (100). Random slopes (of time) and intercepts (of participant) were used for all models. Fixed effects included time (treated as continuous), group, and a group x time interaction. An unstructured covariance matrix was implemented, and standard errors were adjusted via the Satterthwaite correction. Statistical significance was evaluated at a two-sided alpha of *p* < 0.05. To control for Type I Error, a False Discovery Rate (FDR) was applied. Following primary ITT analyses, we examined group differences in reliable change [RCI; (101)] to determine if changes seen in both groups were clinically significant. For these analyses, trajectories of change through unconditional (i.e., random intercept and slope) LMM served as the change score. An RCI of +1.645 is considered a significant change in neuropsychological research, meaning that level of improvement occurs randomly in less than five percent of cases (102, 103). Pearson chi-square was used to test whether groups differed in reliable change.

In a prior randomized controlled trial with participants with PTSD, a medium effect size (*d* = 0.58) was demonstrated in the cognitive rehabilitation group vs. the control (44) on a measure of cognitive flexibility. A power analysis for linear mixed effects models, with three timepoints, *p* < 0.05, power set to 80%, attrition at 20%, and a moderate effect size (*d* = 0.50) indicated that *N* = 144 participants was needed for the present study.

## Results

Descriptive analyses of baseline neurocognitive variables did not show significant differences between treatment groups (all *p* > 0.129; see Table 1). However, there were significantly more participants with TBI in the BHW group compared to the SMART group. Mean scores on all neurocognitive variables, at each time point, for each group, can be found in Table 2. Of participants randomized into the two groups, 97 (75.8%) had at least one follow-up assessment. Pattern-mixture models suggested that missing data patterns did not influence parameter estimates.

**Table 1:** Demographic and baseline characteristics.

**Characteristic**	**Overall**	**SMART** ** (*n* = 66)**	**BHW** ** (*n* = 58)**	***t* or χ^2^**	***p*-value**
Age (*M, SD*)	42.75, 13.67	42.61, 14.14	42.93, 13.20	−0.13	0.90
Male gender (*n*, %)	56, 45.5%	32, 47.8%	24, 42.9%	1.41	0.49
White race (*n*, %)	97, 80.8%	52, 78.8%	45, 77.6%	3.75	0.44
Hispanic ethnicity (*n*, %)	13, 10.7%	10, 15.2%	3, 5.5%	2.99	0.22
Status: number of civilian vs. veteran or military (*n*, %)	68, 60.2%	36, 59.0%	32, 61.5%	0.08	0.79
Bachelor's degree or more education (*n*, %)	57, 47.5%	29, 45.4%	28, 50%	0.26	0.61
PTSD or subthreshold PTSD (*n*, %)	84, 65.6%	39, 59.1%	45, 81.8%	5.59	0.06
Mild or moderate TBI (*n*, %)	117, 95.1%	59, 89.4%	58, 100.0%	5.63	0.02
Mild TBI (*n*, %)	96, 78.05%	49, 75.38%	47, 81.03	0.57	0.45
Moderate TBI (*n*, %)	21, 17.07%	10, 15.38%	11, 18.97%	0.28	0.60
Repeated TBI (*n*, %)	28, 22.95%	13, 20.00%	15, 26.32%	0.69	0.41
Age at first TBI (*M, SD*)	17.71, 10.73	18.72, 10.35	16.68, 11.10	1.03	0.31
Time since last TBI, in years (*M, SD*, range)	10.23, 11.40, 0.00–50.00	9.46, 10.16, 0.00–46.00	11.09, 12.71, 0.00–50.00	−0.77	0.45
Longest LOC in min (*M, SD*, Median)	54.75, 143.34, 10.00	62.26, 167.31, 10.00	46.19, 110.73, 10.00	0.62	0.54
Executive composite score (*M, SD*)	0.03, 1.00	0.02, 1.00	0.04, 1.01	−0.12	0.91
Memory composite score (*M, SD*)	0.02, 1.01	0.00, 1.02	0.04, 1.00	−0.24	0.81
NSI cognitive subscale score (*M, SD*)	6.96, 3.92	6.98, 4.24	6.93, 3.56	0.08	0.94

*PTSD, Posttraumatic stress disorder; TBI, traumatic brain injury; LOC, loss of consciousness; NSI, Neurobehavioral Symptom Inventory*.

**Table 2:** Means and standard deviations of neurocognitive measures by group.

	**SMART**			**BHW**		
**Variable**	**T1**	**T2**	**T3**	**T1**	**T2**	**T3**
CVLT-II trials 1–5 total *T-score* (*M, SD*)	51.18, 10.50	59.69, 12.93	61.11, 12.24	50.50, 11.69	60.11, 11.38	61.50, 12.48
CVLT-II long delay free recall *z-score* (*M, SD*)	−0.03, 0.98	0.67, 0.95	0.71, 0.97	−0.09, 1.28	0.64, 0.98	0.76, 0.99
BVMT-R trials 1–3 total *T-score* (*M, SD*)	43.84, 12.34	49.58, 11.13	51.44, 11.33	44.16, 12.98	52.84, 9.72	52.31, 9.49
BVMT-R long delay free recall *T-score* (*M, SD*)	48.27, 12.29	48.80, 11.06	52.22, 10.24	47.72, 11.17	54.23, 7.59	52.20, 7.87
PASAT total score raw (*M, SD*)	74.88, 24.75	83.65, 23.87	87.86, 18.90	78.81, 21.24	86.20, 20.14	87.21, 24.47
WAIS-IV processing speed index standard score (*M, SD*)	105.30, 14.61	111.08, 13.89	112.04, 13.08	102.89, 13.99	108.95, 15.08	109.79, 17.77
WAIS-IV digit span scaled score (*M, SD*)	11.01, 6.70	10.76, 2.40	10.87, 2.41	10.90, 3.25	11.11, 3.50	11.41, 3.85
WMS-IV logical memory immediate scaled score (*M, SD*)	11.55, 3.17	12.43, 2.82	13.04, 2.62	12.28, 2.55	12.66, 2.45	13.03, 3.00
WMS-IV logical memory delayed scaled score (*M, SD*)	11.51, 3.51	12.86, 3.21	13.44, 3.01	11.90, 3.02	12.98, 2.78	13.41, 3.19
CPT3 omissions *T-score* (*M, SD*)	47.12, 4.45	46.61, 6.75	46.47, 5.20	47.35, 4.83	45.73, 2.62	46.50, 4.53
CPT3 commissions *T-score* (*M, SD*)	50.23, 8.46	46.24, 8.73	46.44, 9.11	50.30, 10.28	47.14, 9.18	45.38, 10.44
VSLT total raw score (*M, SD*)	114.36, 42.81	130.54, 45.95	133.91, 43.70	111.90, 42.21	128.89, 48.07	122.62, 50.10
D-KEFS Number-letter switching scaled score (*M, SD*)	10.68, 2.67	11.67, 2.30	11.64, 2.61	10.84, 2.41	11.66, 2.02	11.88, 2.14
D-KEFS letter fluency scaled score (*M, SD*)	11.16, 3.40	12.53, 3.39	12.67, 3.64	11.53, 3.65	11.50, 3.32	12.15, 4.02
D-KEFS category fluency scaled score (*M, SD*)	11.84, 3.36	10.75, 3.57	12.13, 3.42	11.81, 3.76	10.20, 3.51	11.74, 4.03
D-KEFS inhibition/switch scaled score (*M, SD*)	10.43, 2.99	11.57, 2.62	11.21, 3.47	10.19, 2.94	11.16, 2.84	11.56, 2.78
Executive composite score (*M, SD*)	0.02, 1.00	0.40, 0.96	0.52, 1.02	0.04, 1.01	0.26, 0.95	0.47, 1.20
Memory composite score (*M, SD*)	0.00, 1.02	0.51, 0.98	0.70, 1.01	0.04, 1.00	0.69, 0.93	0.69, 1.00
TOSL summary abstraction score (*M, SD*)	3.00, 1.58	2.92, 1.66	3.10, 1.66	3.09, 1.62	2.68, 1.43	3.23, 1.91
TOSL detail total score (*M, SD*)	8.47, 4.72	10.23, 4.27	9.48, 4.60	8.05, 3.64	9.59, 4.25	9.57, 4.80
TOSL lesson measure–# High (*M, SD*)	0.68, 0.95	0.73, 0.95	0.78,.80	0.47, 0.73	0.57, 0.76	0.49, 0.74

*CVLT, California Verbal Learning Test; BVMT-R, Brief Visual Memory Test–Revised; PASAT, Paced Auditory Serial Addition Test; WAIS, Wechsler Adult Intelligence Scale; WMS, Wechsler Memory Scale; CPT3, Conners' Continuous Performance Test, Third Edition; VSLT, Verbal Selective Learning Task; D-KEFS, Delis–Kaplan Executive Function System; TOSL, Test of Strategic Learning*.

### Principal Components Analysis

Results of the PCA of the neurocognitive variables can be found in Table 3. The parallel analysis identified two components, one of which was comprised primarily of tests of learning/memory and vigilance, whereas the other consisted of tests associated with executive functioning (e.g., abstraction, generativity, mental flexibility/set-shifting, processing speed, inhibition, and working memory). As expected, the components were correlated (*r* = 0.57), which justifies an oblique rotation. All loadings were >|0.40|.

**Table 3:** Principal components analysis of neurocognitive variables.

**Variable**	**Component 1**	**Component 2**
CVLT-II trials 1–5 total	0.60	
CVLT-II long delay free recall	0.47	
BVMT-R trials 1–3 total	0.78	
BVMT-R long delay free recall	0.87	
PASAT total score		0.67
WAIS-IV processing speed index		0.81
WAIS-IV digit span	0.44	
WMS-IV logical memory immediate	0.62	
WMS-IV logical memory delayed	0.66	
CPT3 omissions	−0.52	
CPT3 commissions	−0.50	
VSLT total	0.51	
D-KEFS number-letter switching		0.74
D-KEFS letter fluency		0.78
D-KEFS category fluency		0.67
D-KEFS color-word inhibition/switch		0.68

*CVLT, California Verbal Learning Test; BVMT-R, Brief Visual Memory Test–Revised; PASAT, Paced Auditory Serial Addition Test; WAIS, Wechsler Adult Intelligence Scale; WMS, Wechsler Memory Scale; CPT3, Conners' Continuous Performance Test, Third Edition; VSLT, Verbal Selective Learning Task; D-KEFS, Delis–Kaplan Executive Function System. Component 1 was labeled Memory Composite and Component 2 was labeled Executive Functioning Composite*.

### Trajectories of Change Over Time

Likelihood-ratio tests indicated that linear trajectories of time were appropriate for the data. First, we examined change on the TOSL, the measure of gist learning and the skill directly targeted by SMART. Unconditional growth models indicated that participants did not improve over time on the TOSL summary abstraction score (*b* = −0.02, |*t*| = 0.14, *p* = 0.89) or the high-level lessons score (*b* = 0.02, |*t*| = 0.33, *p* = 0.74). Participants improved significantly over time on the detail total score (*b* = 0.90, |*t*| = 2.32, *p* =.02). Conditional growth models, which tested whether the SMART and BHW groups had differential rates of change over time, were non-significant for the TOSL summary abstraction score (*b* = 0.07, |*t*| = 0.32, *p* = 0.75) the high-level lessons score (*b* = −0.02, |*t*| = 0.19, *p* = 0.85), and the detail total score (*b* = 0.19, |*t*| = 0.25, *p* = 0.81), indicating that the SMART group did not demonstrate superior improvement compared to the BHW group on the TOSL measure.

The results of the unconditional and conditional growth models are summarized in Table 4. Unconditional growth models of the composite scores indicated that there were improvements over time in both domains of cognitive functioning. The overall rate of change in the memory composite indicated that participants improved by 0.31 standard deviation units from baseline to post-assessment on the memory/vigilance composite (|*t*| = 5.23, *p* < 0.001) and by 0.19 standard deviation units in the executive composite (|*t*| = 3.36, *p* = 0.001). Furthermore, participants observed a significant decline in NSI cognitive symptoms (*b* = −0.92, |*t*| = 3.62, *p* < 0.001). Conditional growth models were non-significant for the executive composite (*b* = −0.06, |*t*| = 0.52, *p* = 0.61), the memory composite (*b* = −0.07, |*t*| = 0.62, *p* = 0.53), and the NSI Cognitive subscale (*b* = 0.59, |*t*| = 1.16, *p* = 0.25), indicating that the SMART group was not superior to the BHW group in improving objective cognitive performance or self-report of post-concussive symptoms. We conducted additional analyses separating out the PTSD-only group (*n* = 84) and the TBI-only group (*n* = 117) to assure that the lack of differences was not due to the combined sampling. The pattern of findings remained the same, with no differences between the SMART and BHW group.

**Table 4:** Changes over time by individual neurocognitive tests, for unconditional and conditional growth models.

	**Unconditional**			**Conditional**		
**Variable**	***b* (SE)**	**|*t*|**	***p*-value**	***b* (SE)**	**|*t*|**	***p*-value**
Memory component	0.31 (0.06)	5.23	<0.001	−0.07 (0.12)	0.62	0.53
Executive function component	0.19 (0.06)	3.36	0.001	−0.06 (0.12)	0.52	0.61
NSI cognitive subscale	−0.92 (0.25)	3.62	<0.001	0.59 (0.51)	1.16	0.25
TOSL summary abstraction score	−0.02 (0.11)	0.14	0.89	0.07 (0.23)	0.32	0.75
TOSL lesson measure–# high	0.02 (0.06)	0.33	0.74	−0.02 (0.12)	0.19	0.85
TOSL detail total score	0.90 (0.39)	2.32	0.02	0.19 (0.78)	0.25	0.81

*Unconditional refers to a simple growth model; conditional refers to a group × time interaction*.

Reliable change analyses showed that 61% of the SMART group participants demonstrated clinically significant improvements on the memory composite, compared to 66% of the BHW groups, which did not represent a significant difference (χ^2^ = 0.25, *p* = 0.62). Sixty-seven percent of the SMART group, compared to 64% of the BHW group, showed clinically significant improvements on the executive functioning composite, which did not represent a significant difference (χ^2^ = 0.16, *p* = 0.69). For the TOSL scores, 29% of the SMART group and 33% of the BHW group showed clinically significant improvement on the summary abstraction score (χ^2^ = 0.23, *p* = 0.63), and 58% of the SMART group and 52% of the BHW group showed clinically significant improvement on the detail total score (χ^2^ = 0.43, *p* = 0.51). Twenty-one percent of the SMART group, compared to 9% of the BHW group, showed clinically significant improvement on the TOSL high-level lessons score, a difference which was approaching significance (χ^2^ = 3.77, *p* = 0.05). In addition, groups did not show significant differences in clinically significant improvements on the NSI Cognitive Scale (χ^2^ = 0.70, *p* = 0.40), with 62% of the SMART group compared to 54% of the BHW reporting clinically significant improvement.

### Secondary Analyses

Given that the overall means of baseline memory and EF performance were in the average range, we conducted additional analyses to see if superior effects were observed for SMART when examining only individuals with significant cognitive impairment or moderate TBI. We also sought to verify that the two treatment groups were equivalent on these factors at baseline. First, we examined individuals who scored below 1 SD below the mean on the EF (*n* = 20, 16% of sample) and Memory (*n* = 16; 13% of sample) composites. Twelve participants in the SMART group and 8 participants in the BHW group scored at least 1 SD below the mean on the EF composite, which was not a significant difference (χ^2^ = 0.39, *p* = 0.53). Ten participants in the SMART group and 6 participants in the BHW group scored 1 SD below the mean on the Memory composite, which was also not a significant difference (χ^2^ = 0.64, *p* = 0.43). Examining only participants who scored at least 1 SD below the mean on either of the composites at baseline, LGMMs revealed there to be no significant time, group, or group x time effects on either the EF (*p* = 0.71–0.73) or Memory (*p* = 0.39–0.98) composites.

As noted, participants with moderate TBI performed similarly to those with mild TBI on baseline cognitive measures. We additionally examined potential baseline differences in moderate TBI history for the two treatment groups. Moderate TBI history was not significantly different for the two groups (*n* = 10 in the SMART group, *n* = 11 in the BHW group, χ^2^ = 0.28, *p* = 0.60). We ran LGMM analyses for the moderate TBI group only and did not find any significant time, group, or group × time effects (*p* = 0.73 for memory and.18 for EF) on either the EF or Memory composites. Notably, these subgroup analyses are underpowered, but effect sizes were not suggestive of a different pattern of effects for the subgroups. These additional analyses suggest that the lack of findings between the SMART and BHW groups were likely not attributable to the overall average cognitive functioning of this sample.

## Discussion

This randomized, double-blinded study compared a strategy-based cognitive training program (SMART) to a psychoeducation control group (BHW) in adults with mild and moderate TBI in chronic stages after the injury and/or PTSD. This study builds upon prior research evaluating the effectiveness of SMART by involving a larger sample size of adults with PTSD and/or milder TBI histories and utilizing a shortened, 9-h version of the SMART protocol. Contrary to previous studies demonstrating superior cognitive gains in neurocognitive performance of longer versions of SMART compared to BHW in samples of patients with unclassified TBI with cognitive difficulties (57, 59, 104, 105), we did not observe greater improvements in neurocognitive functioning or gist reasoning in participants enrolled in the shortened SMART protocol. Instead, both groups showed statistically and clinically significant improvements that were maintained over 6 months.

Over 60% of the total sample showed clinically significant improvements on memory and executive functioning, with no significant differences between treatment groups. Memory composite scores improved by over one-half of a standard deviation and executive functioning composite scores improved between one-third and one-half of a standard deviation. Alternate forms were utilized to minimize practice effects on some, but not all, measures. Improvements on specific tests appear to reflect gains beyond practice effects; for example, CVLT total score gains of 1 SD/10-11 words recalled for both groups using alternate forms greatly exceeds published CVLT-II test-retest findings of 1.33 word gains over one month (106). These improvements appear similar to prior SMART studies [e.g., (57, 60)], the main difference being that the BHW group in the present study exhibited similar gains. Both SMART and BHW provide psychoeducation, which has been shown to be an efficacious intervention in the acute phase in reducing self-reported cognitive problems and postconcussive symptoms in patients with mTBI (19, 42, 107), and in improving performance on a measure of attention and information processing speed performance (40). Our results extend this literature to indicate that gains in neurocognitive performance can be seen with psychoeducation alone.

The finding that the BHW group showed similar gains to SMART, contrary to earlier studies (57, 60), suggests that there may be something unique about the current sample, which consisted primarily of individuals with comorbid mTBI and PTSD. Unexpectedly, the BHW group included significantly more participants with TBI histories, and a trend for more participants with PTSD, compared to the SMART group. Although both groups were equivalent in baseline cognitive functioning, the differences in makeup may have contributed to findings. Notably, patients with PTSD and mTBI often hold negative appraisals of their cognitive functioning that are not aligned with their objective neurocognitive performance [(16, 71–74)]. As such, psychoeducation that emphasizes neuroplasticity and implementing simple life changes that can improve brain health may be particularly beneficial for patients who hold negative self-perceptions of their cognitive functioning. This type of psychoeducation may promote self-efficacy, optimism, and hope, allowing patients to believe that change is possible (108, 109). There is evidence to suggest that the perception of cognitive problems, more so than objective neurocognitive functioning, drives functional outcomes related to PTSD (16). As such, simply educating patients with TBI and PTSD about the brain, neuroplasticity, and the ability to improve from injury to the brain has the potential to influence objective cognitive performance as well as functional outcomes.

Alternatively, the unexpected gains observed in the BHW group that were comparable to the SMART group may be due to expectancy effects. Expectancy effects may confound cognitive training study findings (110, 111), particularly when recruitment methods advertise the potential for cognitive gains (e.g., “brain training”). Such methods may lead to a self-selection bias of individuals who expect benefits of the intervention. One experimental study found that participants who were recruited using “overt” flyers advertising a study for cognitive enhancement and describing intelligence gains following working memory training showed significantly stronger improvements on a measure of fluid intelligence after 1 h of training compared to participants recruited with generic flyers (111). Expectations of cognitive gains may influence the motivation to perform better on outcome measures compared to baseline measures (e.g., devoting more effort on memory measures after completing memory training). Similar to prior SMART studies (57), in the current study participants were told that the goal of the study was to compare the benefits of two training programs that could be beneficial for improving cognitive functioning in individuals with histories of head injury or posttraumatic stress. The BHW arm was not described as a control or comparison group and was represented as an active intervention. A major limitation of the study was failure to avoid “overt” recruitment strategies and not assessing for expectancy effects. However, given that recruitment methods were similar to prior SMART studies, these limitations still do not explain the BHW group gains observed in the present study.

There are three primary differences between the current study and prior SMART efficacy studies with TBI: (1) the use of the shortened protocol, (2) inclusion of participants with PTSD, and (3) heterogeneity of TBI history (including longer time since injury) and average baseline cognitive functioning of the sample. As improvements seen in the SMART group appear comparable to prior studies [e.g., (57, 60)], we are hesitant to conclude that the shortened protocol of SMART was “ineffective” because it did not produce superior gains compared to BHW. However, it is possible that studies utilizing the longer protocol or with a sample showing objective cognitive deficits may see greater cognitive gains with SMART. As sustained cognitive deficits following a mild TBI are experienced by a minority of patients [e.g., (4)], it is not surprising that this sample showed average cognitive functioning. It may be that cognitive training is not needed for such individuals, or that greater gains are only observed in individuals with baseline cognitive deficits. We attempted to address this hypothesis by examining the patterns of change in a subset of participants with cognitive deficits (defined as performance <1 SD below the mean) and did not find any group differences in outcomes.

It may be that for clinical populations like mTBI and PTSD, the traditional longer version of SMART is necessary to produce cognitive improvements beyond those seen from psychoeducation. Reinforcing the skills through additional practice may be essential for individuals with clinical conditions. Future studies could examine the benefit of adding continued online training following the in-person group. In addition, we further modified the protocol to replace the typical booster session with additional training. Trainers noted clear emotional and attentional challenges experienced by group members, the majority of whom were struggling with PTSD symptoms that may impair self-regulation and interpersonal functioning. For clinical samples enduring emotional challenges, the longer protocol with a sufficient review may be necessary.

There were a number of limitations to this study that could be improved upon in future research. As noted, cognitive training RCTs should utilize “overt” recruitment methods, removing study-specific information and goals of the study. In addition, assessing expectancy and personal beliefs about the malleability of cognition before randomization would allow for better assessment of training effects and the interaction of expectancy effects with training (111). As participants were recruited from the community, we relied on retrospective self-report of TBI history. Reliable documentation of injury as well as other medical and mental health conditions could improve characterization of a sample. Lacking a more definitive diagnosis of TBI may have contributed to findings. Finally, there are clear limitations to neuropsychological assessment in clinical populations with mild and subtle cognitive deficits like mTBI and PTSD. Neuropsychological assessments may not fully capture the real-world deficits in chronic TBI and PTSD. Testing is performed under idealized, one-on-one conditions, and the subtle impairments experienced by patients may not be adequately captured in that environment. As such, RCTs examining cognitive rehabilitation interventions should include other measures of functional outcomes as well as consider virtual reality functional capacity cognitive assessments.

Overall, we recommend the continued exploration of SMART, a theory-driven, strategy-based, top-down approach to neuroplasticity in mTBI and PTSD samples. Future research investigating both the longer and shortened SMART protocols with patients with PTSD is especially warranted, given the gains observed in this first study examining participants with PTSD. Future researchers undertaking cognitive training RCTs should utilize methods to minimize or tease out expectancy and placebo effects. Finally, researchers should continue to explore the benefits of psychoeducation for individuals with PTSD and TBI, with a focus on assessing potential mechanisms of change.

## Data Availability Statement

The raw data supporting the conclusions of this article will be made available by the authors, without undue reservation.

## Ethics Statement

The studies involving human participants were reviewed and approved by University of Colorado Colorado Springs Institutional Review Board. The patients/participants provided their written informed consent to participate in this study.

## Author Contributions

KS, LB, and CB contributed to conception and design of the study. KS led the project, supervised all study staff, and trained and supervised study clinicians in administration of neuropsychological tests and clinical interviews. KE, AB, TP, MT, and LB managed the study and administered tests and interviews. AB created the database. JJ, KE, and TP performed the statistical analyses. KS wrote the first draft of the manuscript. LA administered the interventions and wrote sections of the manuscript. JJ wrote sections of the manuscript. All authors contributed to manuscript revisions, read, and approved the submitted version.

## Conflict of Interest

The authors declare that the research was conducted in the absence of any commercial or financial relationships that could be construed as a potential conflict of interest.
